# The Usability and Effect of a Novel Intelligent Rehabilitation Exergame System on Quality of Life in Frail Older Adults: Prospective Cohort Study

**DOI:** 10.2196/50669

**Published:** 2025-01-21

**Authors:** Chien-Hsiang Chang, Chun-Chun Wei, Wei-Chih Lien, Tai-Hua Yang, Bo Liu, Yimo Lin, Poh Thong Tan, Yang-Cheng Lin

**Affiliations:** 1 Department of Industrial Design, National Cheng Kung University, Tainan, Taiwan; 2 Department of Creative Design, National Yunlin University of Science and Technology, Yunlin, Taiwan; 3 Department of Physical Medicine and Rehabilitation, National Cheng Kung University Hospital, College of Medicine, National Cheng Kung University, Tainan, Taiwan; 4 Department of Biomedical Engineering, National Cheng Kung University, Tainan, Taiwan

**Keywords:** frailty, exergame, older adults, intelligent rehabilitation, reminiscence therapy, eHealth care

## Abstract

**Background:**

Aging in older adults results in a decline in physical function and quality of daily life. Due to the COVID-19 pandemic, the exercise frequency among older adults decreased, further contributing to frailty. Traditional rehabilitation using repetitive movements tends not to attract older adults to perform independently.

**Objective:**

Intelligent Rehabilitation Exergame System (IRES), a novel retro interactive exergame that incorporates real-time surface electromyography, was developed and evaluated.

**Methods:**

Frail older adults were invited to use the IRES for rehabilitation using lower limb training twice per week for 4 weeks. Participants were required to have no mobility or communication difficulties and be willing to complete the 4-week study. The enrolled cohort had baseline scores ranging from 1 to 5 on the Clinical Frailty Scale, as described by Rockwood et al. Three major lower limb movements (knee extension, plantar flexion, and dorsiflexion) were performed 20 times for each leg within 30 minutes. The surface electromyography collected and analyzed muscle potential signals for review by health care professionals to customize the protocol for the next training. The System Usability Scale (SUS) and Taiwanese version of the EuroQol-5 Dimensions (EQ-5D) were administered after completing the first (week 1, baseline) and last training (week 4, one-month follow-up) to evaluate the usability of the IRES and its effects on the quality of life of participants.

**Results:**

A total of 49 frail older adults (mean age 74.6 years) were included in the analysis. The usability of the IRES improved according to the mean SUS score, from 82.09 (good) at baseline to 87.14 (good+) at 1-month follow-up. The willingness to use (*t*_96_=−4.51; *P*<.001), learnability (*t*_96_=−4.83; *P*<.001), and confidence (*t*_96_=−2.27; *P*=.02) in working with the IRES increased. After using the IRES for 1 month, significant improvements were observed in the ease of use (*t*_47_=2.05; *P*=.04) and confidence (*t*_47_=2.68; *P*<.001) among participants without previous rehabilitation experience. No sex-based differences in the SUS scores were found at baseline or 1-month follow-up. The quality of life, as assessed by the EQ-5D, improved significantly after 1 month of IRES training compared to that at baseline (*t*_96_=6.03; *P*<.001).

**Conclusions:**

The novel IRES proposed in this study received positive feedback from frail older adults. Integrating retro-style exergame training into rehabilitation not only improved their rehabilitation motivation but also increased their learning, system operability, and willingness to continue rehabilitation. The IRES provides an essential tool for the new eHealth care service in the post–COVID-19 era.

## Introduction

Global populations are aging at an escalating speed. The aging-related decline in physical function and quality of life imposes a huge health care burden. Frailty is a clinical aging syndrome attributed to chronic disease interactions and other factors, including an unbalanced diet and adverse lifestyle habits. In Taiwan, the prevalence of frailty and prefrailty in older adults aged 65 years and above is 5.4% and 41.5%, respectively, with a higher prevalence among females than males [1-3]. The clinical presentation of frailty includes decreased activity, abnormal weight loss, reduced appetite, fatigue without apparent cause, muscle loss, gait and balance abnormalities, and cognitive function impairment [4]. Frailty affects the endocrine, cardiovascular, and musculoskeletal systems [5]. Age-related muscle atrophy, known as sarcopenia, correlates highly with frailty [6]. The gait speed of older adults gradually decreases with age and is directly related to the bone and muscle mass of the lower limbs [7]. At present, lower limb rehabilitation involves repetitive training and often does not encourage older adults to perform independently [8,9].

Globally, the predicted demand for health care professionals will reach 80 million in 2030, while the number of available professionals will be less than 18 million [10]. To address this need, the health care system is using innovative technology such as intelligent telemedicine, smart sensor technology, and wearable devices [11,12]. An intelligent health care ecosphere created by information and communications technology is helpful for preventive health care, fitness, and health care services [13]. Interactive technology devices, as effective training tools, improve mobility and balance in patients [14]. Previous studies report significant improvements in several domains of physical function via game-related rehabilitation [15,16]. These findings suggest the effectiveness of exergames, which integrate games into exercise training, to improve physical function during rehabilitation. In addition, mental health is also improved by exergames, which increase the willingness of older adults to participate in long-term exercise [17]. Using exergames for rehabilitation to restore balance in older adults and to shorten the rehabilitation treatment period is an economically beneficial option for home training. Furthermore, exergames with visual feedback provide more effective rehabilitation; such improvement is unlikely to be achieved by traditional manual rehabilitation training, which diminishes with age [18].

Reminiscence therapy, which incorporates life experiences using old photographs or scenarios into activities, is widely used in the care of older adults [19]. This method stimulates long-term memory and aims to maintain patients’ physical and mental health and quality of life, regardless of their cognitive function. The benefits of reminiscence therapy include increasing self-esteem, decreasing depression, and promoting mental health, thereby increasing life satisfaction [20,21].

Older adults are at high risk of COVID-19 infection due to frailty. The COVID-19 pandemic has placed immense pressure on health care systems; in response, a variety of exergames with interactive technology were launched during the pandemic for home use. These exergames can be used as activity tools to maintain muscle strength and decrease physical and mental stress during measures for COVID-19 control [8,9]. In the post–COVID-19 era [22], exergames may help them to keep training and to reduce the burden on the health care system.

In this study, an Intelligent Rehabilitation Exergame System (IRES) that included a retro interactive exergame, real-time surface electromyography (sEMG) used to control the game, and reminiscence therapy was developed for use in the rehabilitation of frail older adults [19,23]. In addition, IRES records and provides data to health care professionals during rehabilitation to allow for the optimization of the rehabilitation protocol [19,23]. The System Usability Scale (SUS) was used to analyze the usability of IRES for rehabilitation [8,24], and the Taiwanese version of the EuroQol-5 Dimensions (EQ-5D) was used to evaluate changes in the quality of life after IRES training in frail older adults [25].

## Methods

### Intelligent Rehabilitation Exergame System

IRES comprises an exergame for rehabilitation that interfaces with a virtual physician, historical photographs with constructed scenarios for reminiscence therapy, and an sEMG for controlling the exergame and recording electrophysiological data for muscle activity during rehabilitation.

### sEMG Signal-Sensing Technology

**Figure 1.**
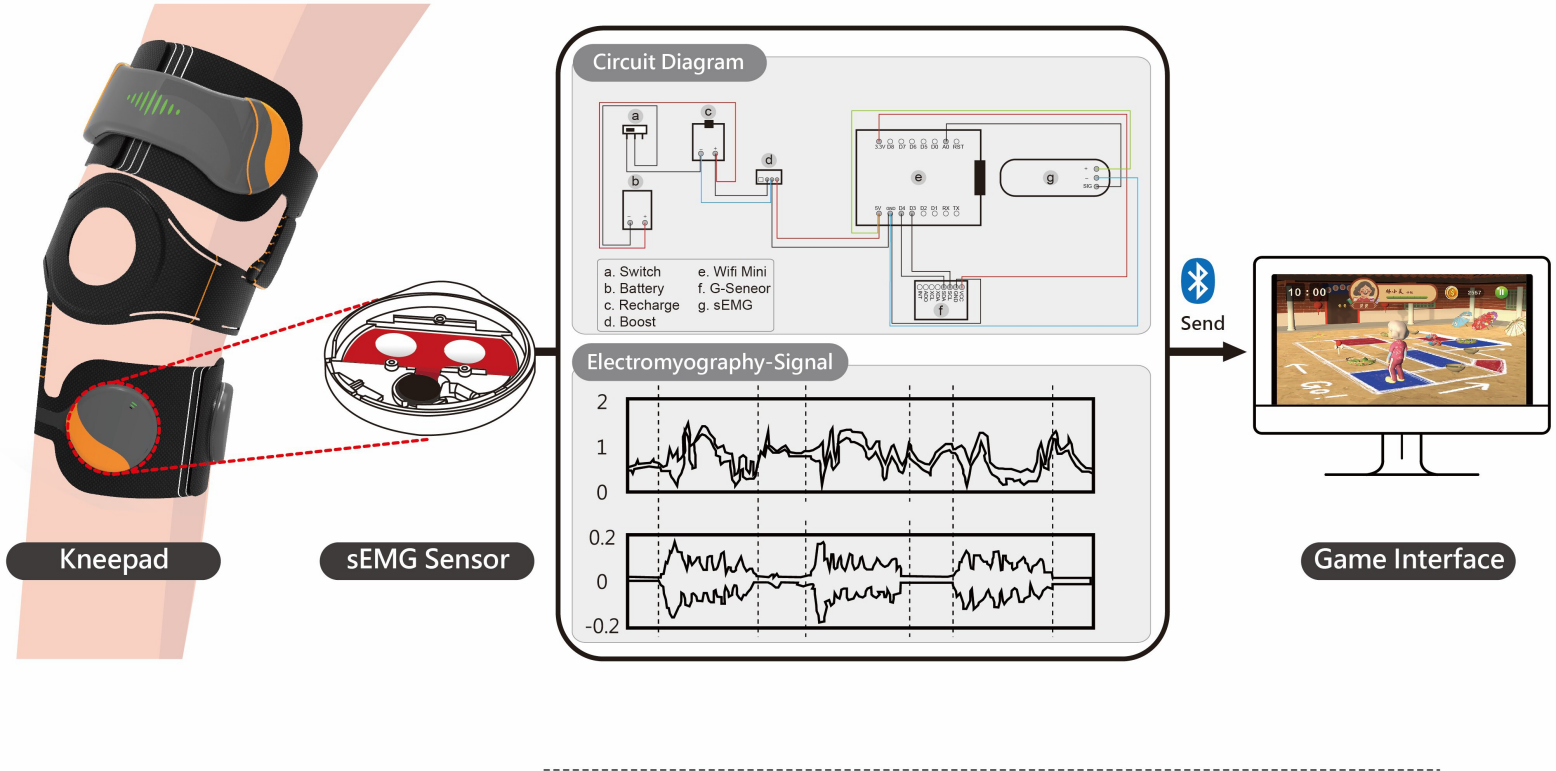
Illustration of the real-time surface electromyography, circuit diagram, and electromyography signal.

sEMG is composed of electrode patches and sensors that are placed on the surface of the skin to detect signals useful for human-machine interactions, gesture recognition, and muscle function evaluation, as previously described [26,27]. Touch fasteners were used to position a kneepad with 3 electrode patches at the bottom of the sensor. The changes in muscle potential signals caused by muscle contractions during limb exercise were measured [28]. The signals were amplified and transmitted by the wireless transmission module to the main unit of the sEMG, then displayed on the screen and stored [27]. The signals were also transmitted to the Unity game software (Unity Technologies), which serves as an interactive multimedia medium to provide visual feedback to the user (Figure 1).

### Design of the Exergame

**Figure 2.**
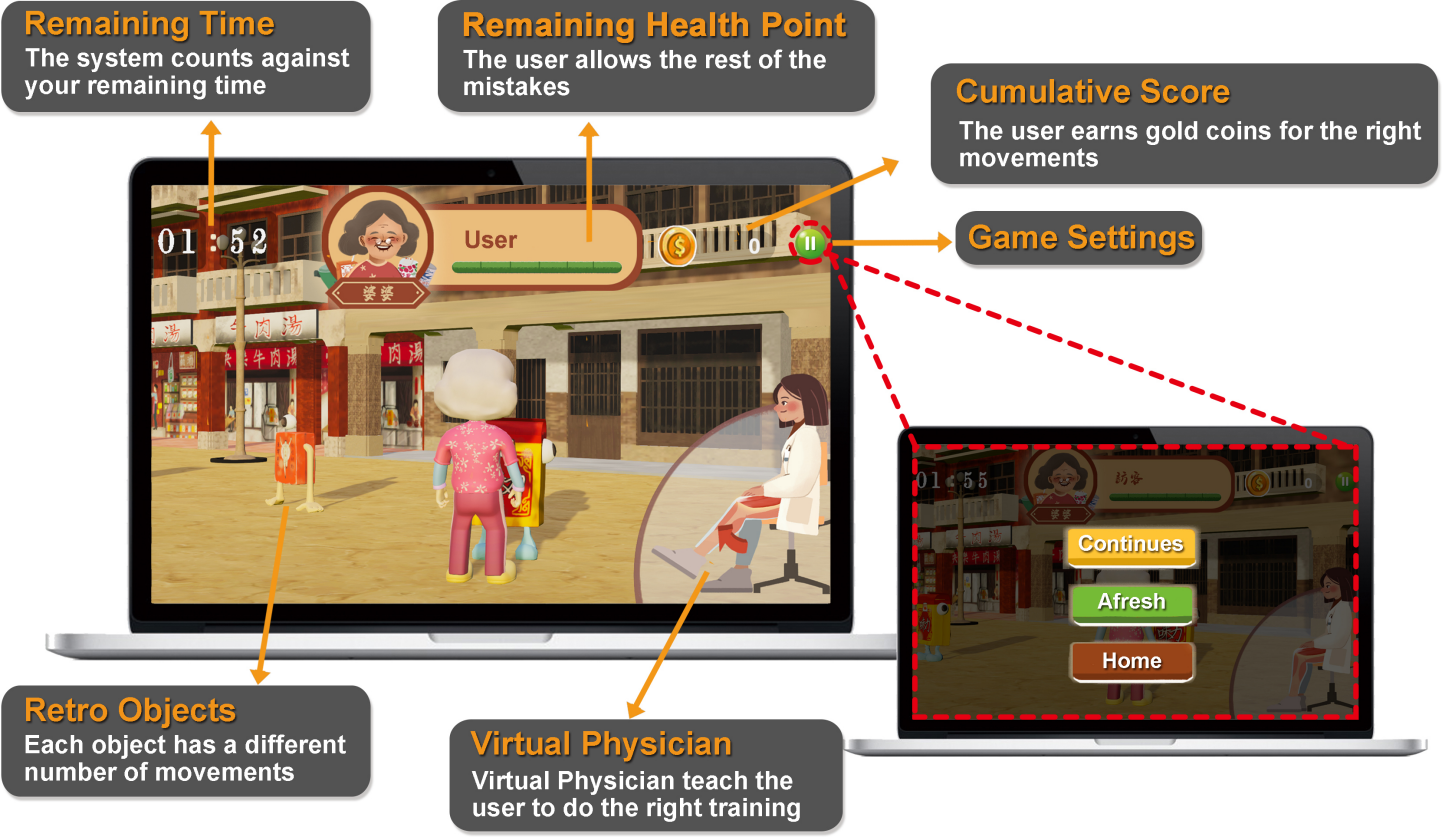
The interface design with the retro style of the IRES (Intelligent Rehabilitation Exergame System).

The IRES system incorporates several innovative features: (1) it is designed to accommodate both medical professionals and frail older adults, (2) it records the sEMG data of muscle group movement during rehabilitation training, (3) this data is then sent for analysis, (4) feedback is provided to medical professionals to assess and adjust rehabilitation parameters, and (5) it recommends the most suitable rehabilitation treatment for the frail older user. Representative historical and cultural images were used for reminiscence therapy that targeted older adults. For example, photographs of the Hayashi Department Store in Tainan in the 1960s were used as the inspiration to draw the interface background, and the interactive components were also designed based on items that were well-known in Taiwan in the 1960s (Figure 2).

**Figure 3.**
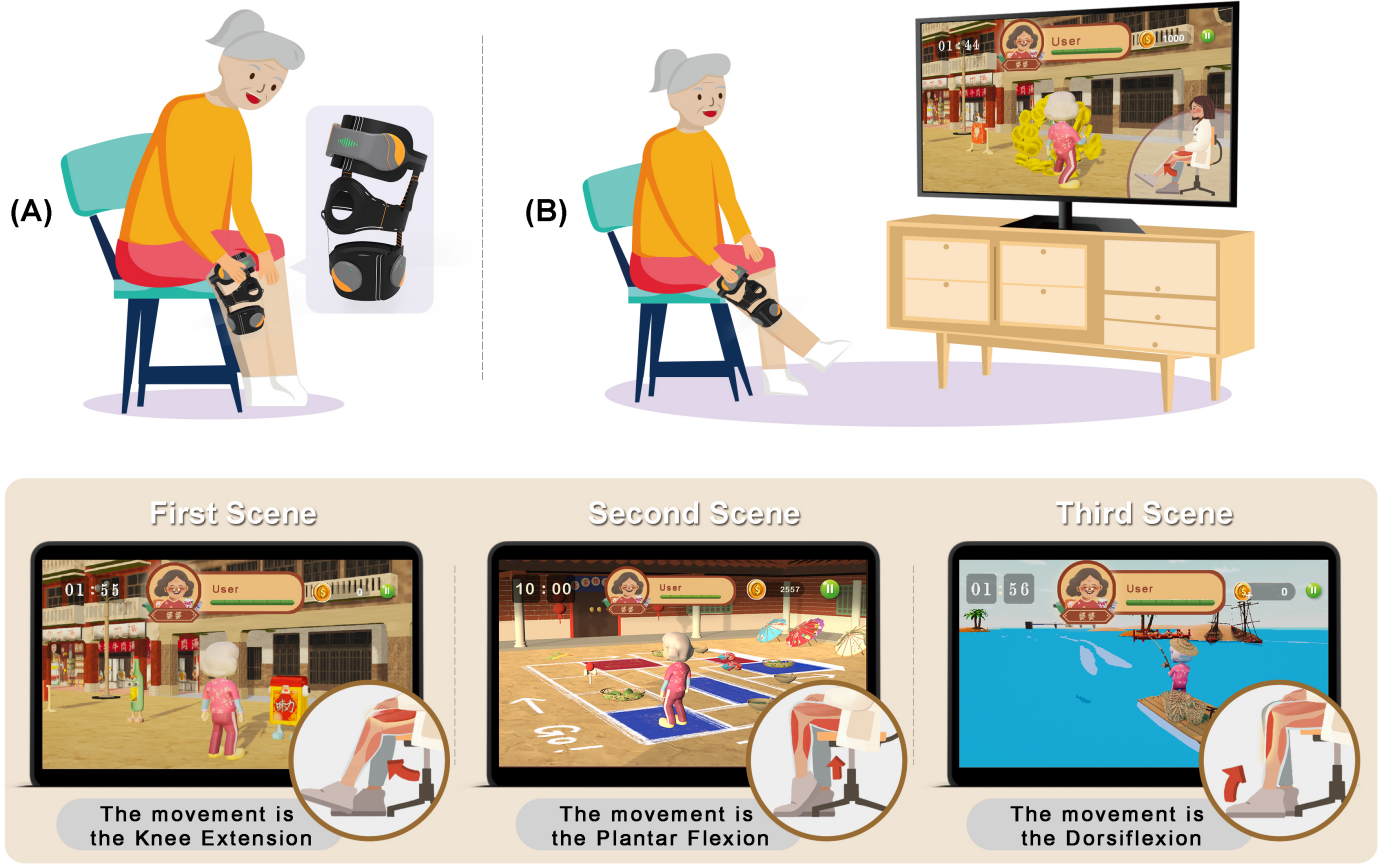
The operation procedure of the IRES (Intelligent Rehabilitation Exergame System): (A) frail older adults wearing the kneepad and placing the surface electromyography sensors and (B) entering the exergame system to perform 3 major rehabilitation movements (ie, knee extension, plantar flexion, and dorsiflexion).

Three scenes were constructed for 3 major lower limb rehabilitation movements—knee extension (Figure 3), ankle plantar flexion, and ankle dorsiflexion for the training of the quadriceps, tibialis anterior, and gastrocnemius muscles, respectively. Participants were able to check the remaining time, remaining health points, and cumulative score on the screen and freely set the exergame to return to the main menu, retry, or continue. The virtual physician was also shown on the screen as a coach to guide and encourage the participants to perform correct movements during training. Misplacement of the kneepad was alerted by the virtual physician (Figure S1 in Multimedia Appendix 1).

### Operation of the Exergame

**Figure 4.**
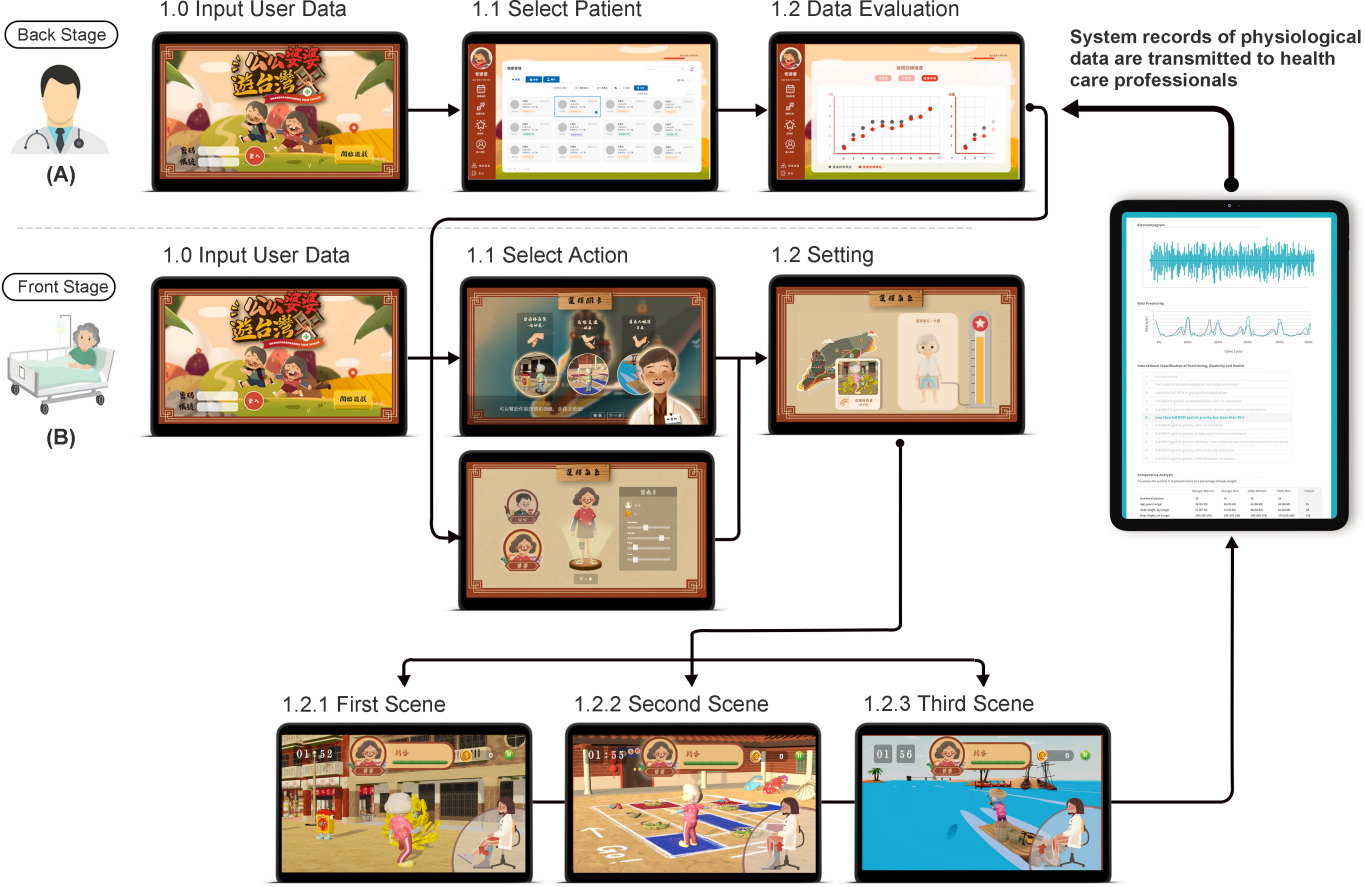
Flowchart for operating the IRES (Intelligent Rehabilitation Exergame System) for (A) health care professionals and (B) frail older adults.

Before the start of the experiment, the researcher explained how the IRES operates and how to accurately wear the kneepad and calibrate the placement of the device. After kneepad placement, the participants logged in to the IRES and selected one scene for training. The first try with maximum strength was used to calibrate the baseline, and then the training began. Each movement was repeated 20 times for each leg within 30 minutes. Training was performed 2 times per week (Monday and Wednesday) for 4 weeks. The training record was analyzed for frequency, intensity, time, and type by the IRES and health care professionals to customize the content for the next training (Figure 4). The environment of the experimental setup is shown in Figure S2 in Multimedia Appendix 1.

### Participant Recruitment

This prospective cohort study was advertised in 2 community centers (Hesing Village and Liren Village, Tainan, Taiwan). Older adults over 60 years old with no mobility and communication difficulties who were willing to complete the 4-week study were enrolled as the inclusion criteria. The exclusion criteria were as follows: (1) unable to walk independently, (2) unable to understand or follow simple instructions, and (3) unwilling to complete the 4-week training.

### Participant Assessment

The Clinical Frailty Scale from 1 (very fit) to 9 (terminally ill) was used to classify the health status of older adults by the physicians at the initial assessment (Table S1 in Multimedia Appendix 1) [29]. Participants assessment was performed after completing the first (week 1, baseline) and the last (week 4, one-month follow-up) training using the SUS for IRES usability [8,24] and the Taiwanese version of the EQ-­5D questionnaire for quality of life [25]. The items included in the SUS are listed in Table S2 in Multimedia Appendix 1.

### Ethical Considerations

This study was approved by the National Cheng Kung University Human Research Ethics Committee (NCKU HREC-F-109-497-2), and the participants provided their written informed consent to participate in this study after receiving a comprehensive explanation of the study from the investigators. All data were deidentified before analysis.

### Statistical Analysis

As previously described by Chang et al [11], at a 95% power and 5% 2-tailed significance level, complete data from at least 15 participants are required to accurately detect differences in the usability analysis. We analyzed power using the G*Power 3.1 software (University of Kiel) [30]. Descriptive statistical data are reported as the mean and SD. Distributions of continuous variables were evaluated using the Shapiro-Wilk test. To assess statistically significant differences in the studied parameters, the independent samples *t* test or Mann-Whitney *U* test was used to compare continuous variables based on the results of the normality tests. The *t* test was used to verify the correlation between usability or EQ-5D and sex or previous rehabilitation experience at baseline and 1-month follow-up. Statistical significance was defined as *P*<.05.

## Results

### Participant Demographics and Change in SUS After IRES Rehabilitation

**Figure 5.**
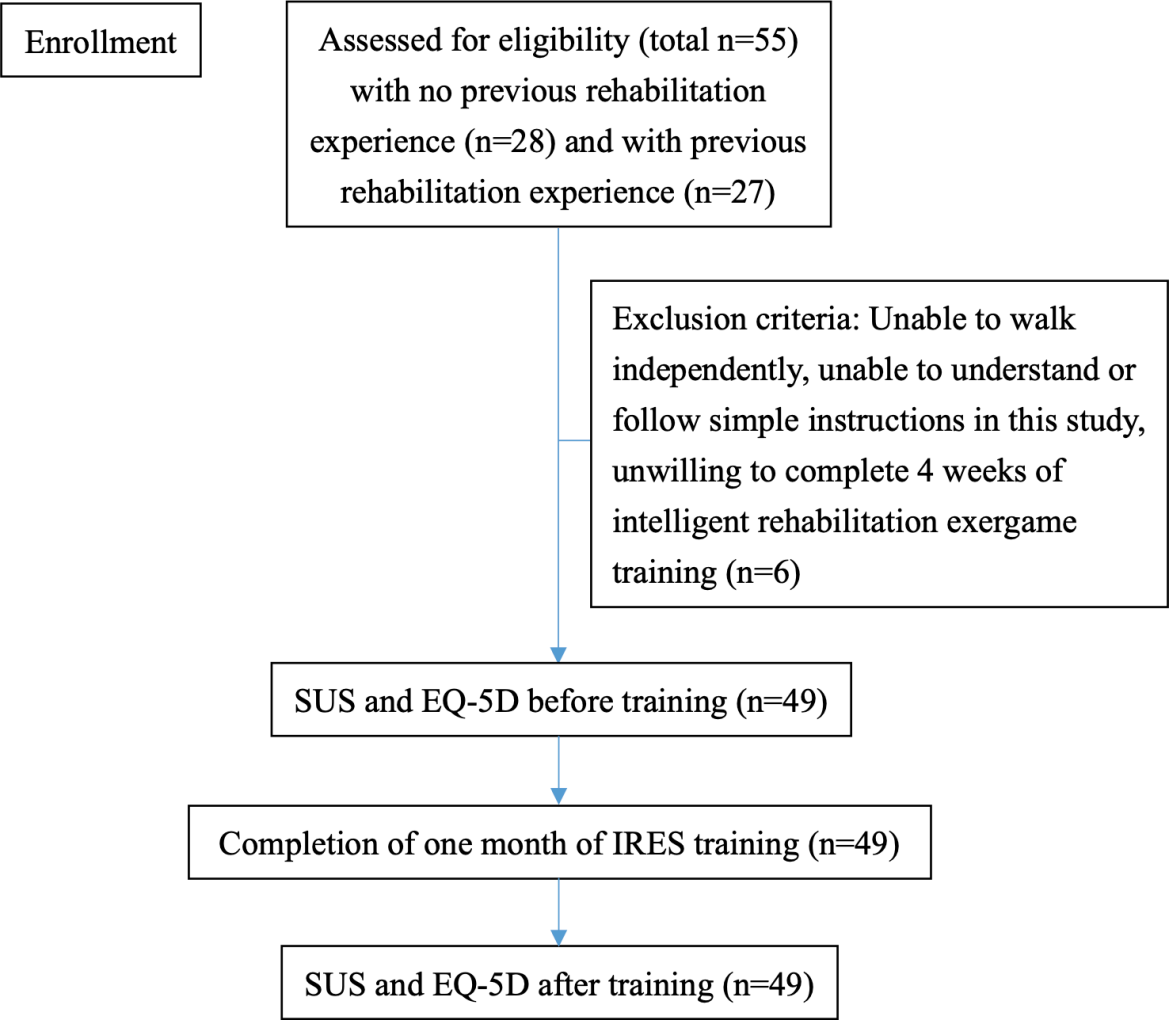
Flowchart for participant recruitment into the study cohort. IRES: Intelligent Rehabilitation Exergame System; SUS: System Usability Scale; EQ-5D: EuroQol-5 Dimensions.

A total of 55 older adults were recruited, including 27 with previous rehabilitation experience and 28 without. Of these participants, 6 were excluded according to the exclusion criteria, leaving 49 participants in the final cohort (Figure 5). The frailty scale scores of the 49 participants ranged from 1 to 5 (score of 1, four participants; score of 2, ten participants; score of 3, twenty-five participants; score of 4, seven participants; and score of 5, three participants). The mean age of the 49 enrolled participants was 74.6 years.

The SUS scores for 10 selected participants at baseline and 1-month follow-up are shown in Table 1. The mean SUS score at baseline was 82.09, with a rating of “good.” At 1-month follow-up, the mean SUS score increased to 87.14 with a rating of “good+.” We observed a significant difference in overall usability between the baseline and the 1-month follow-up (*t*_96_=−2.19; *P*=.03), indicating that the participants felt that the IRES was easy to operate and that its usability increased after 1 month of use.

**Table 1.** SUS^a^ scores for 10 selected participants at baseline (mean 82.09, SD 10.62) and 1-month follow-up (mean 87.14, SD 12.17). A significant difference is observed in overall usability between the baseline and the 1-month follow-up (*t*_96_=−2.19; *P*=.03).

Participant	Sex	CFS^b^	Previous rehabilitation experience	SUS scores	
				Baseline	1-month follow-up
1	Female	5	No	85	72.5
2	Male	3	No	95	80
3	Male	3	No	90	90
4	Male	3	No	92.5	87.5
5	Female	3	No	82.5	97.5
20	Female	4	No	70	95
21	Female	1	No	70	90
22	Female	2	No	87.5	100
23	Female	3	No	100	100
24	Female	4	Yes	80	90
25	Female	2	Yes	92.5	90

a SUS: System Usability Scale.

b CFS: Clinical Frailty Score.

Comparisons of the SUS score between baseline and 1-month follow-up for individual items are shown in Table 2. At baseline, only two items, item 6 (inconsistency) and item 7 (learnability), were “average”; all others achieved “good.” One month after completing the IRES training, item 6 (inconsistency) remained “average,” and the others achieved “good.” The scores increased from “average−” to “average+” for item 6 and from “average” to “good+” for item 7.

**Table 2.** SUS^a^ component and total scores at baseline and 1-month follow-up stratified by previous rehabilitation experience and sex.

Item		Previous rehabilitation experience						Sex						Total (N=49)			Item Benchmark
		Yes (n=26)			No (n=23)			Male (n=12)			Female (n=37)			Mean (SD)	Max	Min	
		Mean (SD)	Max	Min	Mean (SD)	Max	Min	Mean (SD)	Max	Min	Mean (SD)	Max	Min				
1. Willingness																	
Baseline		4.38 (0.69)	5	3	4.52 (0.84)	5	3	4.66 (0.65)	5	3	4.37 (0.79)	5	3	4.44 (0.76)	5	3	Good+
1-month follow-up		4.92 (0.27)	5	4	5.00 (0.00)	5	5	4.91 (0.28)	5	4	4.97 (0.16)	5	4	4.95 (0.19)	5	4	Good+
2. Complexity																	
Baseline		1.84 (1.04)	4	1	1.52 (0.79)	3	1	1.33 (0.88)	4	1	1.81 (0.93)	4	1	1.69 (0.93)	4	1	Good+
1-month follow-up		1.80 (1.41)	5	1	1.43 (1.07)	5	1	1.50 (1.24)	5	1	1.67 (1.29)	5	1	1.63 (1.26)	5	1	Good+
3. Convenience																	
Baseline		4.53 (0.64)	5	3	4.43 (0.84)	5	3	4.50 (0.79)	5	3	4.48 (0.73)	5	3	4.48 (0.73)	5	3	Good+
1-month follow-up		4.34 (0.89)	5	3	4.78 (0.51)	5	3	4.58 (0.79)	5	3	4.54 (0.76)	5	3	4.55 (0.76)	5	3	Good+
4. Stress																	
Baseline		1.11 (0.32)	2	1	1.04 (0.20)	2	1	1.08 (0.28)	2	1	1.08 (0.27)	2	1	1.08 (0.27)	2	1	Good+
1-month follow-up		1.19 (0.80)	5	1	1.34 (1.02)	5	1	1.33 (1.15)	5	1	1.24 (0.83)	5	1	1.26 (0.90)	5	1	Good+
5. Integration																	
Baseline		4.50 (0.64)	5	3	4.56 (0.72)	5	3	4.41 (0.79)	5	3	4.56 (0.64)	5	3	4.53 (0.68)	5	3	Good+
1-month follow-up		4.26 (1.28)	5	1	4.56 (0.99)	5	1	4.75 (0.62)	5	3	4.29 (1.26)	5	1	4.41 (1.15)	5	1	Good+
6. Inconsistency																	
Baseline		2.03 (1.31)	5	1	2.82 (1.52)	5	1	2.25 (1.35)	5	1	2.45 (1.50)	5	1	2.40 (1.45)	5	1	Average–
1-month follow-up		2.07 (1.29)	5	1	2.08 (1.20)	5	1	2.16 (1.19)	5	1	2.05 (1.26)	5	1	2.08 (1.23)	5	1	Average+
7. Learnability																	
Baseline		3.57 (1.33)	5	1	3.86 (1.32)	5	1	3.83 (1.26)	5	2	3.67 (1.35)	5	1	3.71 (1.32)	5	1	Average
1-month follow-up		4.81 (0.49)	5	3	4.69 (0.92)	5	1	4.66 (1.15)	5	1	4.78 (0.53)	5	3	4.75 (0.72)	5	1	Good+
8. Cumbersomeness																	
Baseline		1.69 (0.92)	4	1	1.52 (1.20)	5	1	1.33 (0.88)	4	1	1.70 (1.10)	5	1	1.61 (1.05)	5	1	Good
1-month follow-up		2.11 (1.68)	5	1	1.82 (1.40)	5	1	2.41 (1.83)	5	1	1.83 (1.44)	5	1	1.97 (1.54)	5	1	Good–
9. Confidence																	
Baseline		3.96 (1.03)	5	2	4.30 (1.06)	5	1	4.08 (1.24)	5	1	4.13 (1.00)	5	2	4.12 (1.05)	5	1	Good–
1-month follow-up		4.26 (1.11)	5	1	4.91 (0.28)	5	4	4.50 (1.24)	5	1	4.59 (0.76)	5	2	4.57 (0.88)	5	1	Good+
10. Difficulty																	
Baseline		1.80 (1.13)	5	1	1.56 (0.89)	4	1	1.83 (1.19)	4	1	1.64 (0.97)	5	1	1.69 (1.02)	5	1	Good
1-month follow-up		1.65 (1.19)	5	1	1.26 (0.75)	4	1	1.66 (1.30)	5	1	1.41 (0.92)	5	1	1.46 (1.02)	5	1	Good+

a SUS: System Usability Scale.

### The Effect of Sex and Rehabilitation History on SUS

The effect of sex on differences in SUS scores between baseline and 1-month follow-up was evaluated (Table 3). No significant differences were observed between the sexes for any of the 10 items evaluated (*P*>.05), indicating that the usability of the IRES was consistent between male and female participants.

**Table 3.** Comparison of SUS^a^ scores before and after training according to sex (N=49; 12 men and 37 women).

Item	Baseline		1-month follow-up	
	*t* test (*df*)	*P* value	*t* test (*df*)	*P* value
1. Willingness	−1.13 (47)	.26	0.84 (47)	.4
2. Complexity	1.55 (47)	.12	0.41 (47)	.68
3. Convenience	−0.05 (47)	.95	−0.16 (47)	.86
4. Stress	−0.02 (47)	.98	−0.29 (47)	.76
5. Integration	0.66 (47)	.5	−1.18 (47)	.24
6. Inconsistency	0.42 (47)	.66	−0.27 (47)	.78
7. Learnability	−0.35 (47)	.72	0.48 (47)	.63
8. Cumbersomeness	1.05 (47)	.29	−1.12 (47)	.26
9. Confidence	0.14 (47)	.88	0.31 (47)	.75
10. Difficulty	−0.53 (47)	.59	−0.76 (47)	.44

a SUS: System Usability Scale.

Whether previous rehabilitation experience affected the change in SUS scores between baseline and 1-month follow-up was evaluated (Table 4). At baseline, no significant differences were found between participants with and without rehabilitation experience for any of the 10 items (*P*>.05), indicating that experience does not affect usability. A significant difference between baseline and follow-up was observed for item 3 (convenience: *t*_47_=2.05; *P*=.04) and item 9 (confidence: *t*_47_=2.68; *P*<.001), indicating that IRES use can increase convenience and confidence in older adults without previous rehabilitation experience.

**Table 4.** SUS^a^ component scores before and after training according to previous rehabilitation experience (N=49; yes=26, no=23).

Item	Baseline		1-month follow-up	
	*t* test (*df*)	*P* value	*t* test (*df*)	*P* value
1. Willingness	0.62 (47)	.53	1.35 (47)	.18
2. Complexity	−1.21 (47)	.23	−1.02 (47)	.31
3. Convenience	−0.48 (47)	.62	2.05 (47)	.04^b^
4. Stress	−0.90 (47)	.36	0.59 (47)	.55
5. Integration	0.33 (47)	.74	0.89 (47)	.37
6. Inconsistency	1.94 (47)	.06	0.02 (47)	.97
7. Learnability	0.76 (47)	.44	−0.53 (47)	.59
8. Cumbersomeness	−0.55 (47)	.57	−0.64 (47)	.51
9. Confidence	1.14 (47)	.25	2.68 (47)	<.001^b^
10. Difficulty	−0.82 (47)	.41	−1.35 (47)	.18

a SUS: System Usability Scale.

b Significant difference between groups.

The effect of previous rehabilitation experience on the difference between baseline and 1-month follow-up on each component of the SUS score was analyzed. A significant difference was observed for item 1 (willingness: *t*_96_=−4.51; *P*<.001), item 7 (learnability: *t*_96_=−4.83; *P*<.001), and item 9 (confidence: *t*_96_=−2.27; *P*=.02). Among older adults with previous rehabilitation experience, we observed significant differences in item 1 (willingness: *t*_50_=−3.66; *P*<.001) and item 7 (learnability: *t*_50_=−4.42; *P*<.001). Among older adults without previous rehabilitation experience, significant differences were observed in item 1 (willingness: *t*_44_=−2.71; *P*<.001), item 7 (learnability: *t*_44_=−2.45; *P*=.01), and item 9 (confidence: *t*_44_=−2.65; *P*=.021) (Table 5).

**Table 5.** Changes in SUS^a^ component scores between baseline and 1-month follow-up according to previous rehabilitation experience.

Item	Baseline vs 1-month follow-up					
	Total (N=49)		Previous rehabilitation experience (n=26)		No rehabilitation experience (n=23)	
	*t* test (*df*)	*P* value	*t* test (*df*)	*P* value	*t* test (*df*)	*P* value
1. Willingness	−4.51 (96)	<.001^b^	−3.66 (50)	<.001^b^	−2.71 (44)	<.001^b^
2. Complexity	0.27 (96)	.78	0.11 (50)	.91	0.31 (44)	.75
3. Convenience	−0.40 (96)	.68	0.89 (50)	.37	−1.68 (44)	.09
4. Stress	−1.35 (96)	.17	−0.45 (50)	.65	−1.39 (44)	.17
5. Integration	0.64 (96)	.52	0.81 (50)	.41	0.00 (44)	>.99
6. Inconsistency	1.19 (96)	.23	−0.11 (50)	.91	1.82 (44)	.07
7. Learnability	−4.83 (96)	<.001^b^	−4.42 (50)	<.001^b^	−2.45 (44)	.01^b^
8. Cumbersomeness	−1.37 (96)	.17	−1.12 (50)	.26	−0.79 (44)	.43
9. Confidence	−2.27 (96)	.02^b^	−1.02 (50)	.3	−2.65 (44)	.01^b^
10. Difficulty	1.08 (96)	.28	0.47 (50)	.63	1.24 (44)	.21

a SUS: System Usability Scale.

b Significant difference between baseline and 1-month follow-up.

### The Change in Quality of Life After IRES Rehabilitation

The change in participant quality of life between baseline and 1-month follow-up was evaluated using the Taiwanese version of the EQ-5D questionnaire (Table 6). For the total cohort, all males and all females quality of life improved significantly after the IRES training program (all *P*<.05), indicating a positive effect on the quality of life of older adults regardless of sex.

**Table 6.** Differences in the EQ-5D^a^ between baseline and 1-month follow-up according to sex.

EQ-5D	Total (N=49)		Male (n=12)		Female (n=37)	
	*t* test (*df*)	*P* value	*t* test (*df*)	*P* value	*t* test (*df*)	*P* value
Baseline	0.64 (96)	0.12	0.65 (22)	0.13	0.64 (72)	0.12
1-Month Follow-Up	0.74 (96)	0.1	0.74 (22)	0.11	0.74 (72)	0.1
Total	6.03 (96)	<.001^b^	2.47 (22)	0.03^b^	5.49 (72)	<.001^b^

a EQ-5D: EuroQol-5 Dimensions.

b Significant difference between baseline and 1-month follow-up.

## Discussion

### Principal Findings

We examined the usability of IRES and its effects on the quality of life of frail older adults. The results indicate that IRES increased the willingness of frail older adults to undergo continuous long-term rehabilitation training. The learnability and confidence (ie, self-affirmation) were also higher after 1 month of training compared to those at the baseline. This result indicates that IRES that combines gamified rehabilitation content and movements instructed by a virtual coach can increase the user’s ability to operate and understand the system, thereby decreasing their uncertainty and increasing their confidence in using this novel technology product. In addition, no gender differences were found in the SUS scores between baseline and 1-month follow-up, indicating that both male and female older adults had consistent subjective willingness to use the IRES for the duration of the training. A significant difference was observed in the quality of life between baseline and 1 month after IRES training as assessed by the EQ-5D, further indicating that the quality of life of the participants improved, regardless of their sex.

### The Evolution and Current Trends of Digital Rehabilitation Aids

Rehabilitation aids that combine exercise and games are effective training methods for alleviating frailty and maintaining the health and quality of life of older adults. Such aids also encourage older adults to participate in long-term rehabilitation training, effectively improving physical and mental function while decreasing societal health care burdens [18]. With the maturation and popularization of digital technology, digital games have been used in emerging cognitive training models. Such games, known as serious games, differ from normal entertainment games, as they are purposeful and beneficial to the players. Serious games can encourage older adults to exercise and then increase their physical activity level, further improving muscle strength and cardiopulmonary fitness. Serious games also overcome the low adherence that is generally observed in traditional rehabilitation training, thereby improving the motivation for long-term training [31]. By improving mental focus and increasing muscle mass, exergames can increase the balance, gait, and activity capacity of older adults. Moreover, the integration feature of exergames for older adults provides psychological health benefits [32-34]. The benefit of IRES on the self-esteem and quality of life of the older adults observed in this study likely results from the exergame-based training.

Indeed, information and communications technology and auxiliary life services can effectively improve the life satisfaction of older adults and may increase their willingness to use technology products or services. Considering the larger health care needs of this population, their travel limitations, and health care personnel shortages, intelligent telemedicine care models should be integrated into current health care systems as new tools or services to maintain patient health and prevent disease [35,36]. The following 3 major features need to be incorporated into future health care systems: (1) information and data platform improvements, including the collection and analysis of patient data, immediate provision of individual assistance, and data sharing among medical professionals; (2) health and care services, including the use of virtual and physical communities to provide customized products and care services for patients; and (3) care service support, including supporting and assisting patient care services and promoting health and welfare [36,37]. The appropriate incorporation of intelligent telemedicine can promote the reformation of traditional health care. In the UK National Health Service, the long-term plan is to make intelligent medicine a mainstream practice [38]. However, customized training modules and diverse service content are critical factors proven to affect user acceptance of intelligent telemedicine systems for both patients and health care professionals [39].

### Features and Advantages of the IRES Program Design

The IRES uses gamified training content to increase the pleasure of frail older adults during training, thereby increasing their motivation to continue long-term rehabilitation. In addition, the IRES effectively records, transmits, and analyzes data regarding lower limb muscle movement by sEMG. sEMG measures changes in muscle potential signals that occur when muscles in different motor units are stimulated by motor neurons during limb exercise. The contraction of individual muscles during movements and their potential differences were used to indirectly evaluate individual muscle strength [28]. sEMG is a clinical method widely used in human-machine interactions, hand gesture recognition, and muscle function evaluation. This technology provides data that can be used to evaluate muscle activity and joint movement [26]. These data can be reviewed and studied by health care professionals to evaluate and adjust exercise regimens to provide the most suitable customized rehabilitation treatment for frail older adults.

This feature is another advantage of the IRES over other exergames that are only designed for patients; IRES serves and provides benefits for both patients and health care professionals. Health care professionals could use IRES for continuous interaction and telemedicine services with patients in the post-COVID-19 era [37,40]. Optimization may increase older adults’ acceptance and willingness to continue using this novel product and service, as reported in previous studies [2,6,40]. In the future, IRES-based muscle strength training modules and systems should be developed in medical institutions to help manage health conditions common in frail older adults.

Reminiscence therapy is a people-centered nursing activity that uses patient memories, related past experiences, or specific events [41,42]. Wu et al [43] used old photographs for reminiscence therapy in older adults and found that the photographs significantly evoked autobiographical memories and semantic memory in older adults. Furthermore, reminiscence therapy as a nursing activity improves the sense of self-worth of patients, especially in older adults [44]. Recollecting meaningful people, events, and objects and guiding older adults to discuss past experiences helps them to find meaning in their lives, improves their self-esteem and confidence, alleviates depression, and improves their overall quality of life [45]. Old public photographs are a good format for use in the early care phase of older adults [43]. Integrating these historical images into exergame design can help prevent dementia, apart from providing entertainment. Tsao et al [46] integrated virtual and augmented reality to stimulate memories in older adults while immersing them in retro videos and music environments. These effects are also seen in the results of this study.

### Reception and Effects of the IRES Program

We used the SUS and EQ­-5D to assess the effects of IRES. The SUS is a widely used tool for evaluating the usability of products and systems [24]. After using a product, users are asked to rate their agreement with a series of statements, which are then used to calculate a usability score. The SUS is helpful for developers looking to quickly assess the usability of their product [47]. To correlate clinical information with specific usability criteria in this study, the EQ-5D was used to evaluate older adults’ quality of life. This assessment provides a use value based on the 5D health state classifications: mobility, self-care, usual activities, pain or discomfort, and anxiety or depression. The SUS and EQ­-5D questionnaire results revealed that using a retro style in the exergame design made it very interesting and invoked memories and shared experiences among older adults. This finding is consistent with the results of related studies [11,43,46]. A strong correlation should exist between clinical improvement and the willingness to continue using the rehabilitation system.

A semi-structured interview was also used to gather the participants’ thoughts, willingness to use the IRES, and problems encountered when using it. The results indicate that the retro-style content resonated with older adults, thus improving their willingness to continue using the system. In other words, integrating retro-style content into rehabilitation training could increase the rehabilitation motivation of older adults. The participants reported positive feedback for the IRES system. The functions and treatment course arrangements did not cause adverse events. The IRES system may be suitable for general use among older adults. The human-machine interface design of intuitive interaction and the instructions provided by the virtual coach enabled the system to have better learnability and operability, thus increasing the willingness of older adults to continue rehabilitation.

### Limitations

First, the sample size in this study is relatively small, which limits the generalizability of the results. Further studies using a larger sample size are needed to comprehensively evaluate the usability of the IRES system training in frail older adults. Second, this study has no direct physical function measurement for older adults. In future studies, we would evaluate additional clinical measures such as the strength of the muscle groups used and well-established scales like gait speed, and the timed up-and-go test to investigate the effect on physical function comprehensively. Third, a 1-month training and follow-up may need to be extended in order to achieve greater functional improvement. However, we found a significant improvement in the quality of life for older adults after IRES use. Further long-term studies may be needed to evaluate the long-term effects of IRES system training on frail older adults. Lastly, despite the longitudinal data presented here, the absence of a control group limited a direct comparison to no treatment and quantification of the benefits of IRES, particularly in mitigating physical or cognitive decline over time. The inclusion of a control group should be considered in future long-term studies to validate and extend our findings.

### Conclusion

Population aging and the new normal brought about by the COVID-19 pandemic have increased the importance of frailty and sarcopenia prevention in older adults. Exercise effectively improves fitness, and integrating retro-style exergame training encourages older adults to participate in long-term rehabilitation training to prevent these conditions and decrease the burden on clinics. The results of this study show significant improvements in user willingness and learnability after a 1-month training using IRES. Furthermore, IRES use significantly improved confidence in the use of technology products in frail older adults without previous rehabilitation experience. Overall, IRES not only increased the rehabilitation motivation of frail older adults but also increased their willingness to continue rehabilitation.

## Supplementary material

10.2196/50669 Multimedia Appendix 1 The illustration of IRES and CFS. IRES: Intelligent Rehabilitation Exergame System; CFS: Clinical Frailty Score.

## ABBREVIATIONS

EQ-5D: EuroQol-5 Dimensions
IRES: Intelligent Rehabilitation Exergame System
sEMG: surface electromyography
SUS: System Usability Scale
