# Supplementary material for: The Usability and Effect of a Novel Intelligent Rehabilitation Exergame System on Quality of Life in Frail Older Adults: Prospective Cohort Study
Source: JMIR Serious Games. 2025 Jan 21;13:e50669. doi: 10.2196/50669 (PMC11774325; doi:10.2196/50669)
Supplement: Multimedia Appendix 1 [file games-v13-e50669-s001.docx]

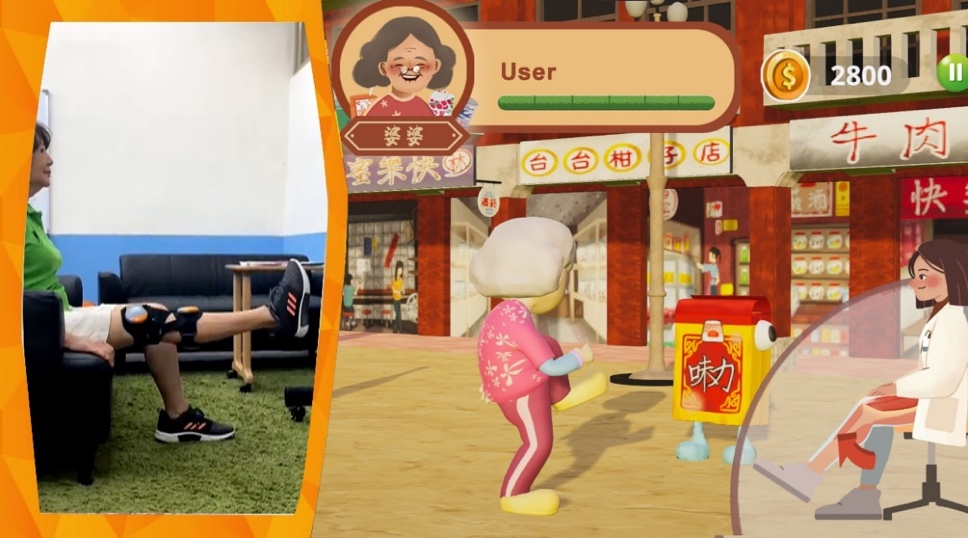


**Figure S1.** The intelligent rehabilitation exergame system (IRES).


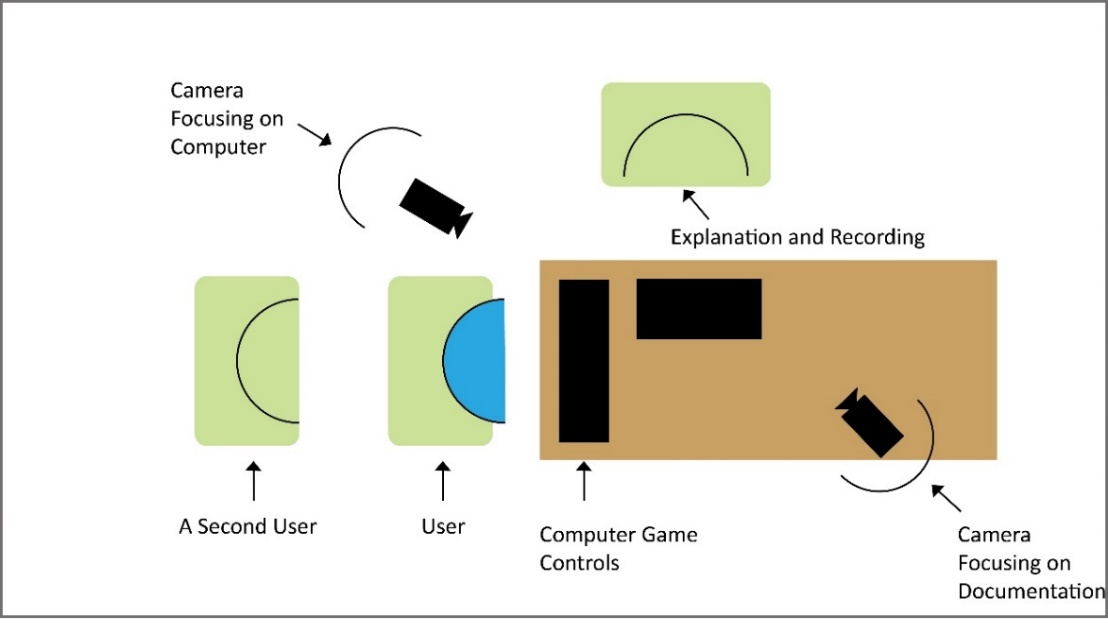


**Figure S2.** The arrangement of the experiment site.

**Table S1.** The clinical frailty scale (CFS).

| 1. Very Fit ^1,2,3,4^ | People who are robust, active, energetic, and motivated and commonly exercise regularly. They are among the fittest for their age. |
| --- | --- |
| 2. Well | People with no active disease symptoms but less fit than category 1. They usually exercise or are very active occasionally (e.g., seasonally). |
| 3. Managing Well | People whose medical problems are well controlled but who are not regularly active beyond routine walking. |
| 4. Vulnerable | While not dependent on others for daily help, symptoms often limit activities. A common complaint is being “slowed up” and/or tired during the day. |
| 5. Mildly Frail | These people often have more evident slowing, and need help in high order IADLs (finances, transportation, heavy housework, medications). Typically, mild frailty progressively impairs shopping and walking outside alone, meal preparation and housework. |
| 6. Moderately Frail | People need help with all outside activities and keeping house. Inside, they often have problems with stairs, and need help with bathing and might need minimal assistance (cuing, standby) with dressing. |
| 7. Severely Frail | Completely dependent on personal care, from whatever cause (physical or cognitive). Even so, they seem stable and not at high risk of dying (within ~6 months). |
| 8. Very Severely Frail | Completely dependent, approaching the end of life. Typically, they could not recover even from a minor illness. |
| 9. Terminally Ill | Approaching the end of life. This category applies to people with a life expectancy < 6 months, who are not otherwise evidently frail. |

**References**

1. Canadian Study on Health & Aging, Revised 2008.

2. K. Rockwood et al. A global clinical measure of fitness and frailty in elderly people. CMAJ. 2005; 173: 489-495.

3. CFS Guidance Document- Version 2020-Apr-06. Geriatric Medicine Research. Dalhousie University.

4. K. Rockwood, O. Theou. Using the clinical frailty scale in allocating scarce health care resources. Can Geriatr J. 2020; 23: 210-215.

**Table S2.** The modified system usability scale questionnaire.

| Item | | Description |
| --- | --- | --- |
| *I1* | *Willingness* | I think that I would like to use the IRES frequently. |
| *I2* | *Complexity* | I find the IRES unnecessarily complex. |
| *I3* | *Convenience* | I think the IRES is easy to use. |
| *I4* | *Stress* | I think that I would need the support of a technical person to be able to use the IRES. |
| *I5* | *Integration* | I find the various functions in the IRES are well integrated. |
| *I6* | *Inconsistency* | I think there is too much inconsistency in the IRES. |
| *I7* | *Learnability* | I would imagine that most people would learn to use the IRES very quickly. |
| *I8* | *Cumbersomeness* | I find the IRES very cumbersome to use. |
| *I9* | *Confidence* | I feel very confident using the IRES. |
| *I10* | *Difficulty* | I need to learn a lot of things before I could get going with the IRES. |
